# Neural reward related-reactions to monetar gains for self and charity are associated with donating behavior in adolescence

**DOI:** 10.1093/scan/nsaa027

**Published:** 2020-03-12

**Authors:** Jochem P Spaans, Sabine Peters, Eveline A Crone

**Affiliations:** 1 Department of Developmental and Educational Psychology, Institute of Psychology, Leiden University, Leiden, The Netherlands; 2 Leiden Institute for Brain and Cognition, Leiden University, Leiden, The Netherlands

**Keywords:** vicarious gaining, prosociality, adolescence, ventral striatum, charity

## Abstract

The aim of the current study was to examine neural signatures of gaining money for self and charity in adolescence. Participants (*N* = 160, aged 11–21) underwent functional magnetic resonance imaging-scanning while performing a zero-sum vicarious reward task in which they could either earn money for themselves at the expense of charity, for a self-chosen charity at the expense of themselves, or for both parties. Afterwards, they could donate money to charity, which we used as a behavioral index of giving. Gaining for self and for both parties resulted in activity in the ventral striatum (specifically in the NAcc), but not gaining for charity. Interestingly, striatal activity when gaining for charity was positively related to individual differences in donation behavior and perspective taking. Dorsal anterior cingulate cortex, insula and precentral gyrus were active when gaining only for self, and temporal-parietal junction when gaining only for charity, relative to gaining for both parties (i.e. under equity deviation). Taken together, these findings show that striatal activity during vicarious gaining for charity depends on levels of perspective taking and predicts future acts of giving to charity. These findings provide insight in the individual differences in the subjective value of prosocial outcomes.

## Introduction

Adolescence is a transitional period between childhood and adulthood, ranging approximately from 10 to 22 years (Crone and Dahl, 2012). This period is characterized by elevated reward sensitivity (Silverman *et al.,* 2015), sensation seeking and a drive to obtain rewards (Steinberg, 2008; Hauser *et al.,* 2015). In addition, a shift takes place from adolescents spending more time outsides the family context, towards spending more time with their peers, resulting in an increasing influence of the peer group on adolescents’ behavior (Steinberg, 2001). Related to this social shift, adolescents develop prosocial goals and social-perspective taking skills to improve (Eisenberg *et al.,* 1995, 2006). These social transitions occur in parallel with an increasing need of adolescents to contribute to society and to develop prosocial goals (Fuligni, 2018). One way to gain more insight into these prosocial motivations is by studying neural activity during prosocial outcomes (e.g. rewards gained for others). In the current study, we examined neural responses to prosocial rewards for charity and how this relates to giving behavior towards charities.

Neuroscientific studies in adults have demonstrated that anticipating rewards (Knutson and Greer, 2008) and gaining rewards for oneself is associated with activity in the ventral striatum, specifically in the nucleus accumbens (NAcc) (Delgado, 2007) and the orbitofrontal cortex (Montague and Berns, 2002; Moll *et al.,* 2006; Berridge and Kringelbach, 2015). Additionally, activity in NAcc correlates with subjective stimulus value (Bartra *et al.,* 2013; Clithero and Rangel, 2014). Activity in the NAcc during reward processing is especially strong in adolescence compared to childhood and adulthood (Galvan *et al.,* 2006; Silverman *et al.,* 2015). This adolescent-specific striatial hyperactivity has been related to negative behavioral outcomes, such as increases in risk-taking behavior (Galvan *et al.,* 2007) and increased alcohol consumption (Braams *et al.,* 2016). Interestingly, whereas early studies have typically related the increase in striatal activity to negative outcomes such as risk-taking (Galvan *et al.,* 2007), more recently studies have nuanced this view by showing associations between increased striatal activity and giving to family in adolescence (Telzer *et al.,* 2013). These findings suggest that whereas adolescence has previously been viewed as a period of risk for negative developmental outcomes, it is possible that adolescence additionally provides rich opportunities for prosocial development (Fuligni, 2018).

An increasing body of evidence shows a parallel between neural activation for self- and other-gains (referred to as vicarious gains). That is, in several studies, vicarious gains were associated with similar NAcc activation as when gaining for self in adults (Morelli *et al.,* 2018; Spaans *et al.,* 2018) and adolescents (Braams and Crone, 2017). Moreover, stronger activity when gaining for friends was associated with everyday prosocial actions in adults (Morelli *et al.,* 2018). Whereas vicarious reward-related activity in the NAcc has been found primarily for vicarious gains for close others, such as family members and friends (Braams *et al.,* 2014b; Braams and Crone, 2017; Morelli *et al.,* 2018), it is not consistently found in all studies (Morelli *et al.,* 2015). Prior research suggested that ventral striatal activity for vicarious gains is dependent on individual differences in willingness to give to others (Kuss *et al.,* 2013), perceived importance of the beneficiary (Telzer *et al.,* 2013; Braams *et al.,* 2014b) and trait empathy (Spaans *et al.,* 2018). Research in adults has demonstrated that the NAcc is involved in gains for charities, which reflects more distal targets (Harbaugh *et al.,* 2007; Genevsky *et al.,* 2013), but it is not yet known how reward-related activity for charity emerges during adolescence. We aimed to explore the processing of these vicarious rewards specifically in adolescence, given its importance as a period of social reorientation (Nelson *et al.,* 2005).

In short, the current study examined neural responses when gaining for self and charity. Previous studies on vicarious rewards used gambling tasks with two outcomes, gains or losses, which preclude the possibility to clearly distinguish between self-loss and other-gain. In the current design, we used a zero-sum false-choice task with a prisoner-dilemma-inspired pay-off scheme, where outcomes could be beneficial to self only, charity only or both parties, relative to a no-gain baseline (previously validated in adults, see Spaans *et al.,* 2018). Given the zero-sum nature of the task, gains for self were always accompanied by no-gains for charity, and vice versa. We decided on using a false-choice task with the aim to decompose the processes (Tamir and Hughes, 2018) underlying vicarious gaining, explicitly focusing on the neural reactions to viewing outcomes for self and charity, and to separate them from decision-making-related activation. Furthermore, we varied the size of the possible outcomes in the task to dissociate absolute and relative amounts of gain (Tabibnia *et al.,* 2008). Based on the literature that suggests that personal gain and vicarious gain have a common neural substrate, we predicted that self-gain, both-gain and charity-gain would all result in activity in the NAcc. Second, based on prior studies showing that closeness to the target might affect whether or not vicarious gains are associated with activity in the NAcc (Telzer *et al.,* 2013; Braams *et al.,* 2014a), we expected charity-gain-related responses to be dependent on self-reported closeness to and perceived importance of the charity. Finally, we addressed the question whether neural responses during vicarious gains predicted individual differences in self-reported perspective-taking, empathy (Spaans *et al.,* 2018) and prosociality as measured by actual donating behavior (Genevsky *et al.,* 2013; Morelli *et al.,* 2018).

## Methods

### Participants and procedure

A total of 160 participants between the ages of 11 and 21 participated in this study (84 females, *M* = 15.99 years, s.d. = 2.95). Three participants were excluded from functional magnetic resonance imaging (fMRI) analyses, two of whom were excluded due to movement during fMRI (>3 mm) and one due to signal dropout in the SPM mask including the ventral striatum (this was assessed by visual inspection of all individual SPM masks). The final analyzed fMRI sample consisted of 157 adolescents. All analyses on behavioral data were conducted with the full sample (*N* = 160). All participants (and their parents if younger than 18) gave written informed consent. All participants were right-handed and had normal or corrected-to-normal vision. Participants were screened with questionnaires on three separate occasions (once by phone-call, once by e-mail and once on the testing-day) for MRI contra-indications and for (history of) neurological and/or psychiatric disorders. All anatomical MRI scans were reviewed by a radiologist. No anomalous findings were reported. The study and all of its procedures were approved by the ethical commission board of the Leiden University Medical Center.

### Materials

#### COSY fMRI-task

**Fig. 1:**
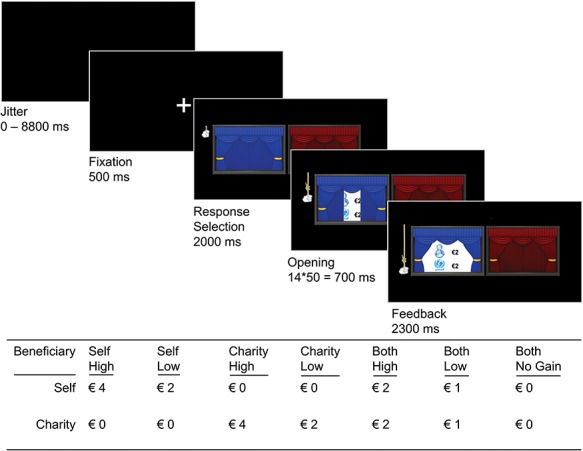
This figure shows the basic trial flow of the zero-sum COSY task. At trial onset, a black screen was presented with a jittered duration between 0 and 8800 ms. Subsequently, a fixation cross was shown for 500 ms, followed by the response selection screen for 2000 ms. After a response was made, an animation was shown onscreen for the remainder of the 2000 ms. Then, the next 14 screens showed a fluid animation of the hand pulling the curtain open and revealing the outcome (shown here; self €2, charity €2). The feedback remained onscreen for 2300 ms. In case participants failed to respond within the timeframe of the response selection, no animation occurred and a screen with the phrase ‘Too Late!’ was shown for 3000 ms. Outcome conditions are displayed in the table below the trial flow.

To investigate responses to vicarious gains for charity, we used a false-choice fMRI-task called the charity or self-yield (COSY) task, which we used in an earlier study with a separate adult sample (Spaans *et al.,* 2018). In the COSY task, participants can earn money for themselves and for a previously self-chosen charity (see Supplementary File A for the list of charities) by deciding which out of two curtains to open on every trial. After participants press a button, an onscreen hand indicates what option they have selected. Next, the chosen curtain opens in a fluid animation (14 frames presented for 50 ms each), with the outcomes fully visible from the seventh frame onwards. The outcomes were either a division of 4 Euro stakes between parties, or a division of 2 Euro stakes between parties. All outcomes were zero-sum, with gains for one party being inversely related to gains of the other party. Specifically, in case of a division of 4 Euros (high magnitude), this could result in the following outcomes: self high [€4 self, €0 charity]; charity high [€0 self, €4 charity] or both high [both €2]. In case of a division of 2 Euros (low magnitude), this could result in the following outcomes: self low [€2 self, €0 charity]; charity low [€0 self, €2 charity: charity low] or both low [both €1]. In both conditions, these outcomes were contrasted against a zero gain baseline both no gain [both €0]. The two stakes were used to control for magnitude when examining the effects of mutual gain (Tabibnia *et al.,* 2008). A black jitter screen (0–8800 ms) was presented after the outcome presentation, marking the end of a trial (see Figure 1 for a graphical representation of the trial flow and the outcome conditions).

Every outcome condition occurred 15 times during the task, resulting in a total of 105 trials. The order of trials was optimized for our design using the program Optseq2 (Dale, 1999). The task consisted of two blocks of 50 and 55 trials, respectively. Each block lasted ~6 min. At the end of the research day, participants and charity received extra money for completing the COSY task (both charity and the participant could earn €1–2 in steps of €0.50, rewards were counterbalanced across participants).

#### Questionnaires

##### COSY manipulation checks

After the fMRI session, participants answered several questions about the COSY-task. First, to obtain a subjective measure of the enjoyment when gaining for charity, we asked how it felt to win different amounts of money (€0, €1, €2 and €4) for self and charity, answering on a scale where 1 = ‘did not feel good at all’ and 7 = ‘felt very good’. Second, we asked whether participants thought they could influence the outcome and why they thought this was or was not the case. We also asked participants how important they rated the charity, and how well they knew what the charity stood for. All of these questions were answered on seven point Likert scales. Finally, we asked participants whether or not they normally donated to this charity in daily life (1 = yes, 2 = no, 3 = sometimes), and whether they thought they could influence the outcome of the task (1 = yes, 2 = no, 3 = sometimes).

All charities were chosen at least once by the participants. The perceived importance of chosen charities was high for all participants (*M* = 5.84, s.d. = 1.14), and participants reported to have knowledge of the charity (*M* = 4.58, s.d. = 1.36). There was a significant correlation between perceived importance of the charity and knowledge about the charity (*r*(157) = 0.35, *P* < 0.001). To the question whether participants donated in daily life, 85 participants answered ‘yes’ or ‘sometimes,’ and one-hundred-and-two answered ‘no.’ Finally, to the question whether participants believed they could influence the outcome, 10 participants answered ‘yes,’ one-hundred-and-two answered ‘no’ and 45 answered ‘sometimes.’ We checked whether individual differences in the perception of being able to influence the outcomes during the task were associated with differences in neural activity in the task, or to variations in donation behavior. A set of ANOVAs showed this was not the case (all *P* > 0.68).

##### Empathy and perspective taking

To investigate individual differences in empathy and perspective taking, we included the empathy and perspective taking subscales of the interpersonal reactivity index (IRI) questionnaire (Davis, 1980; Hawk *et al.,* 2013). Both subscales were reliable, with Chronbach’s alpha values of respectively 0.72 and 0.79.

##### Behavioral donating task

In the exit interview after the fMRI session, participants played a one-shot dictator game. That is, they were given the choice to distribute 600 valuable coins (unbeknownst to participants, 100 coins were worth €0.50, for a total of €3) between themselves and the charity of their choice by selecting one of seven possible divisions on a scale (1 = 600 for self, 0 for charity; 2 = 500 for self, 100 for charity; 3 = 400 for self, 200 for charity; 4 = 300 for self, 300 for charity; 5 = 200 for self; 400 for charity; 6 = 100 for self; 500 for charity; 7 = 0 for self and 600 for charity). In order to prevent socially desirable behavior, it was stressed that their chosen distribution would remain completely anonymous. To ensure anonymity, the final amount displayed on the screen for the experimenter was a sum of the money gained in the fMRI task and the money divided in the one-shot dictator game. In total, participants could earn a range of €1–5 extra for themselves and charity, depending on their donation decisions.

#### MRI data acquisition

MRI data were acquired using a Philips 3.0 Tesla scanner with a standard whole-head coil attached. For functional MRI scans, we used T2*—weighted Echo-Planar Imaging (TR = 2.2 s, TE = 30 ms, FOV: 220 × 220 × 111.65 mm, voxel size = 2.75 × 2.75 × 2.75). Functional scans consisted of two runs with 175 and 169 volumes, respectively. Participants were able to see the screen through a mirror that was attached to the head coil. The functional task lasted for ~13 min in total. In addition to fMRI sequences, we collected structural images for anatomical reference (high-resolution 3D T1), TR = 9.751 ms, TE = 4.59 ms and FOV = 224 × 177 × 168 mm. Participants’ head movements were restricted by using foam triangles to limit available space in the coil.

#### MRI data analyses

##### Preprocessing

We used the software package SPM8 (Wellcome Trust Centre for Neuroimaging, London) to preprocess and analyze all MRI-data. For preprocessing, we first corrected all MRI-images for motion (runs with any framewise displacement motion higher than 3 mm were excluded), corrected them for slice timing acquisition and consequently spatially normalized the functional scans to T1 templates. Then, all volumes were resampled to voxels of 3 × 3 × 3 mm. We based our templates on the MNI305 stereotaxic space (Cocosco *et al.*, 1997). Finally, we used an isotropic Gaussian Kernel (6-mm FWHM) to spatially smooth the data.

##### fMRI-analysis

To calculate the relevant contrasts, we modeled the fMRI time series convolved with the hemodynamic response function with events that corresponded to the outcome phase of a trial. Specifically, the events of interest that we modeled were the outcome conditions ‘self high,’ ‘self low,’ ‘charity high,’ ‘charity low,’ ‘both high,’ ‘both low’ and ‘both no gain.’ These events were time-locked with zero-duration to the exact moment that participants were able to see the outcome; the seventh frame of the curtain-opening animation. Trials with no response from the participants were coded as ‘missing’ and modeled separately as invalid trials, and were not included in further contrasts. The modeled events were added as regressors in a general linear model, along with a motion regressors and a basic set of cosine functions that high-pass filtered the data and a covariate for session effects. The least-squares parameter estimates of height of the best-fitting canonical HRF for each condition were used in pairwise contrasts. The resulting contrast images, computed on a subject-by-subject basis, were submitted to random-effects group analyses. Contrast analyses for each beneficiary (self, both and charity) relative to both no gain were performed using *t*-tests. Effects of beneficiary (self, both and charity) and magnitude (high and low) were examined in a 3 × 2 full factorial whole brain ANOVA in SPM. All images were thresholded by using a false discovery rate (FDR) cluster correction (initial threshold at *P* < 0.001). For a visual representation of the activation in these contrasts *vs* the fixation baseline, see Supplementary Figure B1 (Supplementary File B). For the time series of activation in these contrasts after stimulus onset and feedback onset, respectively, see Supplementary Figures C1 and C2 (Supplementary File C).

##### fMRI region-of-interest analysis

To investigate the effects that emerged in after our initial whole brain analyses ANOVA, we performed the region-of-interest (ROI) analyses using the Marsbar toolbox (Brett *et al.,* 2002).

In addition, we tested a priori hypotheses about reward-related activity on a predefined anatomical ROI (Braams *et al.*, 2015) of the left and right NAcc, extracted from the Harvard–Oxford subcortical atlas and thresholded at 40%. The mask consists of 28 voxels for the left NAcc (center-of-mass coordinates left: *x* = −9.57, *y* = 11.70, *z* = −7.10) and of 26 voxels for the right NAcc (coordinates right: *x* = 9.45, *y* = 12.60, *z* = −6.69). We extracted parameter estimates for the ROI analyses. Since no significant differences in activation were found between left NAcc and right NAcc, all were consequently performed by collapsing across the left and right hemispheres of the NAcc.

All reported results are available on NeuroVault (Gorgolewski *et al.,* 2015), see https://neurovault.org/collections/UNRMPFBJ/.

#### Analysis plan

##### Behavioral analyses

To test whether the fMRI task was effective in eliciting subjective reward, we investigated the behavioral enjoyment ratings made by participants after the fMRI task. To this end, we performed a 4 (magnitude) × 2 (beneficiary: self or charity) repeated measures ANOVA and performed planned comparisons to follow up the significant main within-person effects. Next, to explore the distribution of donation behavior and to check whether there were differences in donation behavior for different charities, we respectively computed means and standard deviations of donation behavior and conducted an ANOVA with chosen charity as independent variable and donation as dependent variable.

##### Analyses of neural activation

The analyses for neural activity in the vicarious reward task were conducted in two steps. First, we performed whole brain analyses to test for effects of condition, using whole-brain contrasts. Second, we performed ROI analyses on the anatomical NAcc to test for the a priori hypothesized relations with donation behavior.

Specifically, to investigate the relationships between ROI activation in NAcc and donation behavior, we conducted repeated measures ANCOVAs in SPSS with beneficiary as factor (three levels: self, both and charity) and, empathy, perspective taking and donating behavior as covariates in separate analyses to examine if patterns differed depending on variation in these variables.

Lastly, we exploratively conducted a 3 × 2 whole-brain ANOVA with beneficiary (three levels: self, both and charity) and magnitude (high and low) as factors, and performed the follow-up analyses on ROI extracted from the regions that emerged from the whole brain analyses ANOVA (see Table 4 for coordinates) using the Marsbar Toolbox (Brett *et al.,* 2002).

## Results

### Behavioral results

#### Enjoyment ratings

**Fig. 2:**
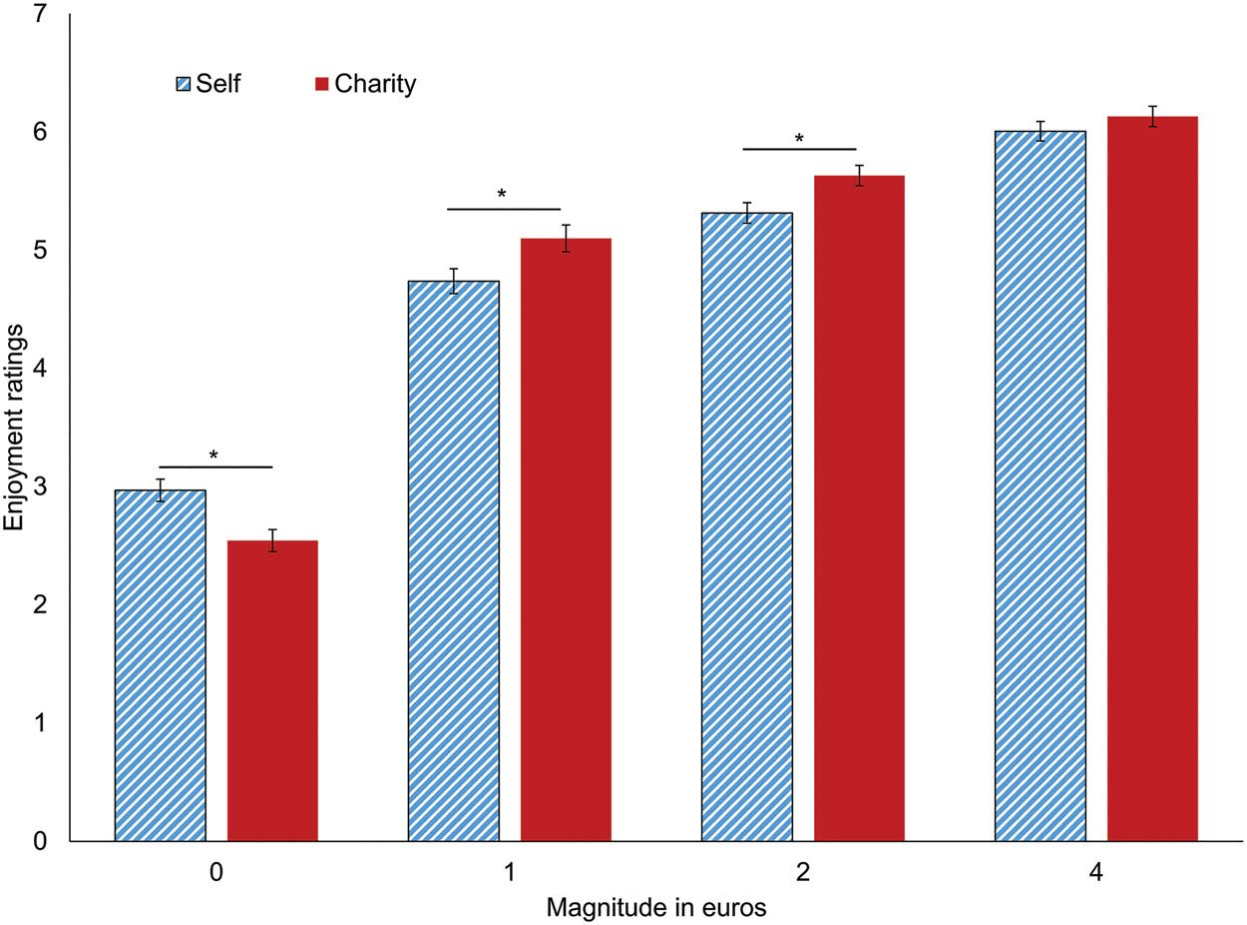
Bar chart representing winning enjoyment for different beneficiaries and magnitudes. Enjoyment is plotted on the *y*-axis (scale ranged from 1 to 7) and different sets of bars represent different magnitudes (€0, €1, €2, €4). Blue-colored bars represent outcomes for self, red-colored bars represent outcomes for charity. Differences significant at *P* < 0.05 are flagged with ‘*’.

We tested whether the task was effective in eliciting subjective reward by examining subjective enjoyment ratings for different magnitudes of reward (€0, €1, €2 and €4) and for different targets (self, charity, both self and charity). Results showed a main effect of Magnitude (*F*(3, 477) = 480.79, *P* < 0.001, ${\eta}_{\mathrm{p}}^2$= 0.75) and a magnitude by beneficiary interaction (*F*(3, 477) = 12.99, *P* < 0.001, ${\eta}_{\mathrm{p}}^2$= 0.08) (see Figure 2). The latter interaction showed that for both beneficiaries, higher magnitudes were rated as more enjoyable (€4 < €2 < €1 < €0; all differences *P* < 0.001). However, within-magnitude comparisons showed that participants enjoyed it less when charity received nothing compared to when they themselves received nothing (*F*(1, 159) = 14.31, *P* < 0.001), and enjoyed it more when, compared to self, charity received 1 euro (*F*(1, 59) = 13.11, *P* < 0.001) or 2 euros (*F*(1, 159) = 11.70, *P* < 0.001). For 4 euros, there was no differences between beneficiaries (*F*(1, 59) = 1.29, *P* = 0.26).

#### Behavioral donating task

**Fig. 3:**
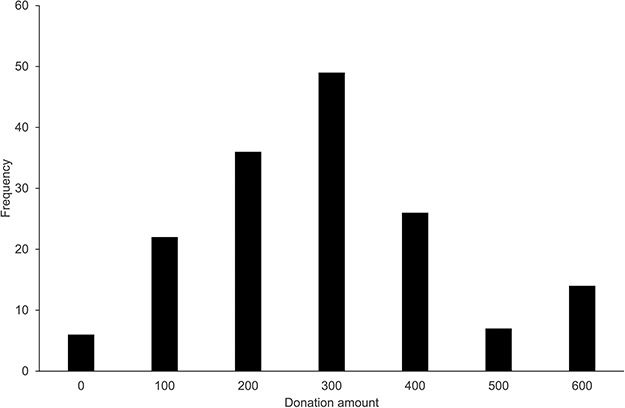
This figure shows the absolute frequencies of prosocial donations made in the one-shot dictator game. Participants could divide 600 valuable tokens by picking one of seven divisions.

Next, we addressed the question how much participants donated in the one-shot dictator game and how this related to individual ratings of importance, knowledge about charity, perspective taking, empathy and age. Participants donated on average 247 of the 600 coins to charity (s.d. = 127.64). There were no significant differences in donation amount between charities (*F*(9, 150) = 2.25, *P* = 0.44, ${\eta}_{\mathrm{p}}^2$ = 0.057). See Figure 3 for a histogram displaying the frequency of each donation.

Donating behavior was negatively correlated to how much participants reported enjoying gaining for self (average of 1, 2 and 4 euros) (*r*(160) = −0.364, *P* < 0.001) and positively correlated to how much participants reported enjoying gaining for charity (average of 1, 2 and 4 euros) (*r*(160) = 0.234, *P* < 0.001) and to self-reported importance of the chosen charity (*r*(160) = 0.191, *P* = 0.016).

There was no correlation between donating behavior and age, self-reported empathy, self-reported perspective taking or self-reported knowledge about the chosen charity (see Table 1 for a full overview of correlations). As can be seen in Table 1, age was positively correlated with perspective taking (*r*(160) = 0.31, *P* < 0.001) and negatively correlated with the importance rating of the charity (*r*(160) = −0.29, *P* < 0.001). All other correlations are also presented in Table 1.

**Table 1:** Correlations between self-reported empathic concern (IRI-EC), perspective taking (IRI-PT), enjoyment of self-gains and charity gains, importance of charity, knowledge of charity, donation behavior in the donating task and age

	Perspective taking	Self-gains	Charity-gains	Importance	Knowledge	Donation behavior	Age
Empathic concern	0.307^***^	−0.026	0.030	0.264^**^	0.108	0.118	−0.103
Perspective taking		−0.082	−0.041	−0.097	−0.026	0.061	0.306^***^
Self-gains			0.430^***^	0.046	0.026	−0.364^***^	−0.049
Charity-gains				0.271^**^	0.198^**^	0.234^**^	−0.102
Importance					0.350^***^	0.191^*^	−0.290^***^
Knowledge						0.066	−0.084
Donation behavior							0.129

Degrees of freedom = 160. Significant correlations are flagged at *P* < 0.05 with ^‘*’^, *P* < 0.01 with ^‘**’^ and at *P* < 0.001 with ^‘***’^. See Supplementary Figure E1 for a version of this table with written out *P*-values.

### Neural activity

#### Gain conditions *vs* no-gain

**Fig. 4:**
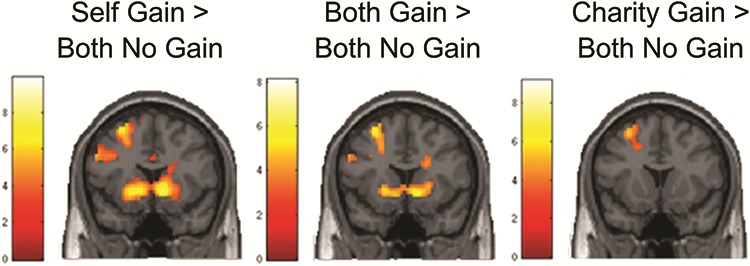
From left to right: Respective activation patterns in the NAcc for self gain > both no gain, both gain > both no gain and charity gain > both no gain contrasts. Coronal view at coordinates *y* = 12. Activation displayed is FDR cluster corrected (initial threshold *P* < 0.001, accepted thresholds, respectively, 273 for self gain > both no gain, 63 for both gain > both no gain and 173 for charity gain > both no gain).

First, to test whether NAcc activity was observed for the three beneficiary conditions, relative to a no gain baseline (in which both beneficiaries receive €0), we computed whole brain contrasts for each condition. As can be seen in Figure 4, for the contrast self gain > both no gain and both gain > both no gain, activity was observed in NAcc, but not for charity gain > both no gain. All other activations are reported in Table 2.

**Table 2:** Coordinates for contrasts self gain > both no gain, charity gain > both no gain and both gain > both no gain

Contrast	Region	*T*	*K*	*x*	*y*	*z*
Self gain > both no gain	Anterior cingulum	9.92	3332	0	47	7
	Medial orbital gyrus	8.93		0	53	−5
	Right caudate	7.71		12	14	−2
	Precuneus	7.65	1905	−6	−64	37
	Frontal superior medial gyrus	6.83		0	−40	31
	Left angular gyrus	6.41		−36	−67	46
	Right precentral gyrus	5.28	523	18	−31	67
	Right postcentral gyrus	5.12		30	−31	70
	Right precentral gyrus	5.01		42	−22	58
	Left paracentral lobule	4.98	273	−9	−34	70
	Left precentral gyrus	4.45		−27	−25	64
	Left postcentral gyrus	4.31		−36	−31	58
Char gain > both no gain	Precuneus	9.18	1963	3	−58	31
	Left angular gyrus	7.59		−51	−67	28
	Frontal medial orbital gyrus	7.64	1488	0	59	−2
	Left frontal superior medial gyrus	7.35		−9	65	22
	Left frontal superior gyrus	6.52		−21	29	49
	Right angular gyrus	5.79	173	57	−61	28
Both gain > both no gain	Cuneus	8.10	1173	−3	−64	25
	Precuneus	7.32		−6	−58	16
	Middle cingulum	6.19		−6	−43	37
	Left occipital midline	6.36	362	−36	−73	40
	Anterior cingulum	6.27	369	0	41	7
	Left frontal superior gyrus	6.26	978	−21	29	46
	Right caudate	5.92		9	17	−5
	Left caudate/putamen	5.36		−15	11	−5
	Right postcentral gyrus	6.25	746	21	−34	64
	Right parietal superior gyrus	5.41		21	−49	67
	Right precentral gyrus	5.18		21	−25	73
	Left frontal inferior triangle	5.72	291	−45	29	19
	Left parietal superior gyrus	5.45	307	−21	−46	70
	Paracentral lobule	4.29		0	−28	58
	Left paracentral lobule	3.94		−6	−34	70
	Right temporal superior gyrus	4.88	273	60	−13	7

FDR cluster corrected (initial threshold *P* < 0.001), accepted FDR thresholds were 273 (self gain > both no gain), 173 (charity gain > both no gain) and 63 (both gain > both no gain). AAL atlas was used for labeling. Subclusters that fell outside of defined regions in the AAL are not included in this table. For cluster sizes under 600 voxels, here we present only the main cluster coordinates. See https://neurovault.org/collections/UNRMPFBJ/ for the full overview of activation.

#### Neural activity in the NAcc and donation behavior

Next, we examined correlations between donation behavior and neural activity in an independent anatomically defined ROI of the NAcc. We averaged across magnitudes as there was neither main effect of magnitude nor an interaction with magnitude in our whole brain analyses for the NAcc (see section Exploratory analyses).

We found a negative correlation between activation in the self gain > both no gain contrast and donation behavior (see Table 3). More specifically, participants with relatively higher activity in the striatum when gaining for self, donated less to charity. Finally, to check whether activation did not differ depending on the charity participants chose, we checked whether neural activity in the self gain > both no gain, both gain > both no gain and charity gain > both no gain contrasts differed depending on the chosen charity. This was not the case (all *P* > 0.53).

**Table 3:** Correlations between age, donation behavior and NAcc activation in self gain–both no gain, both gain–both no gain and charity gain–both no gain

	Donation behavior	NAcc self gain	NAcc both gain	NAcc charity gain
Age	0.129	−0.177^*^	−0.161^*^	−0.044
Donation behavior		−0.175^*^	−0.068	0.102
NAcc self gain			0.625^**^	0.537^**^
NAcc both gain				0.615^**^

Degrees of freedom = 160. Significant correlations are flagged at *P* < 0.05 with ‘*’ and at *P* < 0.001 with ‘**.’ See Supplementary Figure E2 for a version of this table with written out *P*-values.

Next, we tested if these variables showed interactions with individual differences in repeated measures ANCOVAs with beneficiary as factor (three levels: self, both and charity) and empathy, perspective taking and donating behavior as respective covariates. This resulted in two significant interactions. For donating behavior, there was a significant beneficiary × donating interaction, *F*(2, 316) = 8.42, *P* < 0.001, ${\eta}_{\mathrm{p}}^2$ = 0.051. For perspective taking, there was a significant beneficiary × perspective taking interactions, *F*(2, 316) = 3.22, *P* = 0.019, ${\eta}_{\mathrm{p}}^2$ = 0.025. No interactions were found for empathy.

**Fig. 5:**
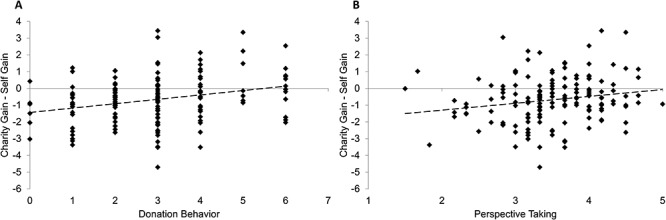
Relations are displayed between activation in charity gain—self gain and donation behavior (A) and charity gain—self gain and perspective taking (B).

To further investigate these patterns, we subtracted charity gain from self gain to obtain a difference score that reflects the degree of similarity between the neural activity for charity and self-gains. Note that given the zero-sum nature of the game, charity-gains were accompanied by the absence of gains for self, and self-gains were accompanied by the absence of gains for charity. As a result, in this difference score, a score of 0 on this difference score indicates identical activity for self and charity, whereas negative and positive scores, respectively, reflect a stronger striatal activation to either self-gains compared to no-gains for charity, or charity-gains compared to no-gains for self. We found that this difference score was significantly correlated to perspective taking (*r* = 0.19, *P* = 0.012) and donating (*r* = 0.279, *P* < 0.001) (Figure 5A and B). Thus, more perspective taking and more donating were both significantly correlated to more charity-*vs*-self-related NAcc activity. No correlations were found for both gain–self gain.

#### Exploratory analyses

Lastly, we conducted a whole-brain ANOVA to investigate the effects of beneficiary and magnitude. Results showed a significant main effect of beneficiary with eight significant clusters, no significant effect of magnitude and a significant beneficiary by magnitude interaction with one significant cluster (see Table 4 for cluster coordinates). Main effects of beneficiary were observed in bilateral NAcc, bilateral insula/inferior frontal gyrus, dorsal anterior cingulate cortex (dACC), precentral gyrus and bilateral temporal-parietal junction (TPJ).

**Table 4:** Coordinates for the main effect of beneficiary and beneficiary by magnitude interaction for the 3 × 2 ANOVA whole-brain ANOVA

Contrast	Region	*F*	*K*	*x*		*y*	*z*
Main effect beneficiary	Right NAcc	24.97	145	12		11	−2
	Right frontal inferior	11.40		15		2	−8
	Right insula	18.68		33		20	−11
		16.38		42		23	−8
	Supplementary motor area	20.07	1613	12		11	61
	Right supplementary motor area	19.63		9		20	58
	Left anterior cingulum	18.49		−3		38	25
	Left NAcc	19.15	114	−9		8	−2
	Right angular gyrus	18.59	182	60		−55	31
	Right temporal midline	7.23		60		−58	16
	Left frontal midline	17.43	140	−24		50	28
	Left frontal superior gyrus	14.21		−27		59	22
		10.22		−36		53	13
	Left insula	15.47	152	−42		17	−2
	Left angular gyrus	11.49	56	−57		−58	34
	Right frontal midline	10.62	54	45		11	46
Interaction beneficiary × magnitude	Anterior cingulum (mPFC)			−3		47	10
		14.99	429				
		12.26		6		47	4
		11.24		9		41	16

Analyses were FDR cluster corrected (initial threshold *P* < 0.001), accepted thresholds 53 (main effect beneficiary) and 429 (interaction beneficiary × magnitude). AAL atlas was used for labeling. See https://neurovault.org/collections/UNRMPFBJ/ for a full overview of activation.

**Fig. 6:**
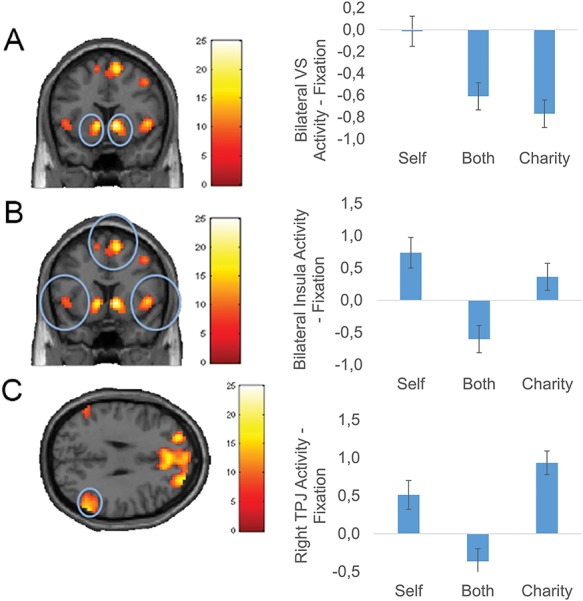
Displayed are the patterns of activation for the main effect of beneficiary in (A) bilateral NAcc *vs* the fixation baseline (*y* = 10), (B) in bilateral insula *vs* the fixation baseline (*y* = 10) and (C) in right TPJ *vs* the fixation baseline (*z* = 30). Activation was collapsed over magnitudes for the different beneficiaries. With respect to the activation in bilateral insula (B), similar patterns were observed in dACC and precentral gyrus. Due to the zero-sum nature of the task, self gain means no gain for charity, and charity gain means no gain for self. Activation displayed is FDR cluster corrected (initial threshold *P* < 0.001, accepted threshold 80). Correlates of activation in ROIs derived from this contrast with age can be found in Supplementary File F.

Visual inspection of the activation in each region and subsequent *post hoc* testing revealed three general patterns (see Figure 6A–C). First, the activity pattern in bilateral NAcc corresponded to the amount gained for self, showing largest activity in the self gain condition, followed by the both gain condition, followed by the charity gain condition (Figure 6A; all *P*’s < 0.05). Second, bilateral insula, dACC and precentral gyrus were more active for self gain and charity gain than both gain (with relatively more activity for self gain compared to charity gain; all *P*’s < 0.05), potentially indicating that these regions respond when outcomes were different from equity (Figure 6B displays bilateral insula only; dACC and precentral gyrus showed similar patterns). In addition, right TPJ was also more active when outcomes were different from equity, that is for self gain and charity gain, with relatively more activity for charity-gains compared to self-gains (Figure 6C; all *P*’s < 0.05). The pattern was similar for left TPJ, except activation for self and charity did not significantly differ from each other.

**Fig. 7:**
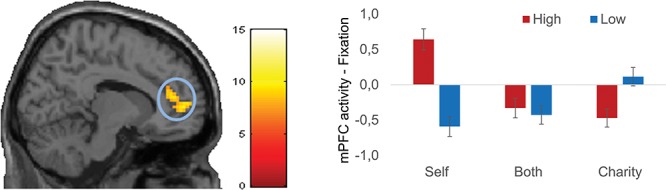
The target × magnitude interaction in mPFC is displayed in the figure (*x* = 0). Different bars display activation for different targets. Blue bars represent low-magnitude gains, whereas red bars represent high-magnitude gains. Due to the zero-sum nature of the task, self gain mean no gain for charity and charity gain mean no gain for self. Activation displayed is FDR cluster corrected (initial threshold *P* < 0.001, accepted threshold 429).

The beneficiary × magnitude interaction resulted in a cluster in the medial prefrontal cortex (mPFC). To investigate these effects in more detail, we examined this pattern in a ROI extracted from this region. As can be seen in Figure 7, this region was more active for low charity-gains than for high charity-gains. In contrast, this region was more active for high self-gains compared to low self-gains. No difference between magnitudes was observed for the both conditions.

## Discussion

The aims of the current study were 2-fold. First, we aimed to test the neural responses observed in the NAcc during vicarious gains for charity in adolescence, and compare them to self-gains and gains for both parties. In addition, we exploratively tested the involvement of other regions in vicarious gaining.

Second, we examined the relations between activity in the striatum during (vicarious) gains for self and charity, and prosociality, with a specific focus on individual differences in empathy, perspective taking, self-reported enjoyment, relationship with the charity and donation behavior.

### Neural correlates of gaining for self and charity

#### NAcc

First, we investigated whether NAcc activity was dependent on whether gains were experienced for self or charity. Consistent with prior studies comparing self- *vs* other-gains (Montague and Berns, 2002; Delgado, 2007; Berridge and Kringelbach, 2015), the NAcc was more active when participants gained money for themselves (at the expense of charity). Interestingly, we found increased activity in the NAcc when gains were obtained for both self and charity (both gain condition), but not when gains were for charity only (at the expense of self).

This supports the notion that the NAcc reflects the valuation of uniquely self-relevant outcomes. That is, monetary outcomes for self were on average highest in the self conditions (for a mean self-gain of €3.00), followed by the both conditions (for a mean self-gain of €1.50), followed by the charity conditions (for a mean self-gain of €0.00). As such, the most parsimonious explanation would be that responses in the NAcc covaried with the absolute outcomes for self, regardless of gains for charity (Morelli *et al.,* 2015).

Surprisingly, we found no significant whole brain effect of magnitude in the NAcc. Given the role of the NAcc in subjective valuation, we would have expected activation in this region to covary with increases in gain-amount. Possibly, since activation was most pronounced in the self conditions, having the both and charity conditions included in this whole brain analysis washes out the main effect of magnitude. The magnitude × target analyses did result in significant activation in mPFC, supporting the notion that participants did differentiate between magnitudes in the task.

#### Medial prefrontal cortex

The mPFC, a brain region often co-activated with the NAcc in reward processing (Van Duijvenvoorde *et al.,* 2016) and associated with self-relevant processing (Denny *et al.,* 2012), showed a significant beneficiary by magnitude interaction. Specifically, mPFC showed increased activation relative to all other conditions when gaining high magnitudes for self and when gaining low magnitudes for charity. Possibly, the mPFC tracks subjective significance of outcomes (Schmitz and Johnson, 2007). Gaining high magnitudes for self may be highly salient for participants, whereas low rather than high charity outcomes may be experienced as most subjectively salient given that the relative loss for self is low, while at the same time this could be considered a prosocial outcome. These findings fit with the subjective behavior ratings for which participants indicated that they enjoyed gaining 2 euros slightly more for charity than for self. Future studies should follow up these results, but possibly the mPFC is involved in monitoring the value of rewards received by oneself vs others (Dal Monte *et al.,* 2018).

#### Temporal-parietal junction

The right TPJ showed higher activation in self- and charity-conditions compared to both-conditions and was more active for charity than self. Activation in TPJ has often been related to perspective taking abilities (Blakemore and Mills, 2014) or switching between perspectives of self and others (Carter and Huettel, 2013; Schurz *et al.,* 2014). A recent study found that TPJ recruitment was higher when adolescents passively viewed prosocial scenes compared to social and noninteractive scenes (Tashjian *et al.,* 2018). These findings align with the finding of stronger TPJ recruitment for charitable giving. Future studies should examine the specificity of TPJ for prosocial motivations.

The activity in mPFC and TPJ is in line with previous research on the roles of these regions in mentalizing about others’ thoughts and intentions (Frith and Frith, 2003; Burnett *et al.,* 2009; Van Overwalle, 2009), and with studies that show that activity in mPFC is related to the subjective valuation of donations (Hare *et al.,* 2010; Bartra *et al.,* 2013; Clithero and Rangel, 2014).

#### Salience network

A separate network of brain regions seemed specifically sensitive to gains that benefited one target, relative to gains for both parties. Specifically, the bilateral insula, dACC and the precentral gyrus showed highest activation for outcomes for self only, followed by charity only, with least activation in the condition which benefited both. These regions are all part of the salience network (Seeley *et al.,* 2007; Sridharan *et al.,* 2008; Menon and Uddin, 2010), and have been found to be active for highly self-relevant, salient information (Perini *et al.,* 2018). One possible interpretation for higher saliency in these conditions is that they include higher absolute pay-off amounts per target (€2 and €4 *vs* €1 and €2 euro’s), and as such might have drawn more attention. Moreover, prior studies have also shown that deviation from the equity norm (an equal allocation between targets) is experienced as a salient event, and is associated with increased activation in dACC and insula (Güroğlu *et al.,* 2014).

### Gaining for self and charity and prosociality

The second goal of this study was to relate vicarious reward responses in a false choice paradigm, where outcomes were controlled, to actual prosociality in terms of behavior and self-reports. Here, we found that activity in the striatum for vicarious charity-gains was related to individual differences in donation behavior, self-reported enjoyment and perspective taking.

Interestingly, donation behavior outside the MRI scanner was related to the difference in activity found for charity- and self-gains during the fMRI task. Increased activity for charity-gains compared to self-gains was related to higher donations.. However, given the zero-sum nature of the current paradigm (where gains for self imply less gains for charity), it should be noted that interpretations about personal rewards are unequivocally linked to the absence of vicarious rewards, and vice versa.

These findings on the relationship between activation during vicarious gaining and donation behavior are consistent with prior research in adults, which reported that vicarious activity in the NAcc for gains for friends was associated with real life prosociality (Morelli *et al.,* 2018). Here, we observed similar findings for self-reported enjoyment for self- and charity-gains. That is, we found that the self-reported enjoyment of the respective gains for self and charity was significantly related to donation behavior. Together, these findings show that activity in the striatum during vicarious gaining reflects the valuation of prosocial outcomes, and that it relates to tendencies to perform prosocial behavior (Moll *et al.,* 2006; Harbaugh *et al.,* 2007; Genevsky *et al.,* 2013). Additionally, the current findings show that perspective taking may be an important factor that underlies vicarious reward responses to charity, as individuals who scored higher on perspective taking also showed stronger reward-related activity in the NAcc when vicariously gaining for charity.

Given that adolescence might be a period in which individual differences in prosociality emerge, an interesting question concerns whether or not prosociality of adolescents is prone to change as a result of interventions or community programs. A meta-analysis on community service programs demonstrated that positive effects of community service on prosocial values were dependent on whether the program involved reflection (Van Goethem *et al.,* 2014). Given that community programs with reflection elements can affect prosocial values, an interesting question for future research is whether fostering these values may also lead individuals to experience stronger vicarious reward for unknown others.

Finally, there were some unexpected findings in the current study that should be addressed in future research. In self-report, participants indicated that they enjoyed gaining for charity more than gaining for themselves. They also reported that they preferred losses for themselves over losses for charity. However, if striatal activity reflects processes related to the valuation of an outcome, one would expect the self-reported enjoyment for charity-gains to be correlated to the striatal activity for charity-gains, which was not the case in the current study. Perhaps, self-reported enjoyment is more subjected to social desirability bias than the neural activity. Participants may be prone to overstate their reported subjective enjoyment for charity-gains, as being charitable is a socially desirable characteristic. Related to this, the propensity to be sensitive to demand characteristics could explain the unexpectedly high enjoyment ratings for charity-gains. Possibly, participants might have felt that answering in a prosocial way (by reporting high charity-gain enjoyment or even by donating high amounts of tokens in the one-shot dictator game) was appropriate or expected of them. We tried to limit the role of possible demand effects by stressing that there were no right or wrong answers, by masking the outcome of the one-shot dictator game, by having several different experimenters, and by refraining from communicating the purpose of this part of the study. However, latent demand propensities cannot be ruled out as a possible confound with the current paradigm. Possibly, similar processes (e.g. implicit social norms) could play a role in daily life donating as well (Martin and Randal, 2008). Finally, reporting how you feel about winning afterwards and online neural activation during the task could reflect complementary processes of valuation. These seemingly contradicting findings warrant further investigation.

### Limitations and future directions

The current study had some limitations that should be addressed in the future research. First, the design made use of a false-choice paradigm to control the number of gain events. This, however, limited the possibility to relate the neural activity to actual task choice. Future studies should therefore extend these findings by also including choice paradigms (Moll *et al.,* 2006; Harbaugh *et al.,* 2007; Genevsky *et al.,* 2013). Second, since we measured importance to a charitable organization rather than actual relationship closeness, direct comparisons against previous studies that did gauge relationship closeness in vicarious gaining paradigms should be interpreted with caution. Possibly, importance of a charity is related differently to individual differences in valuation of vicarious gains than relationship closeness to a stranger, friend or family member. Finally, the current study was cross-sectional, whereas developmental relations, such as between perspective taking, prosocial valuing and giving behavior in adolescence, can best be studied using longitudinal designs (Taris, 2000; Telzer *et al.,* 2018).

## Conclusions

Taken together, the current study confirmed that in adolescence vicarious reward activity in the NAcc when gaining for charity is significantly related to subsequent prosocial behavior. This neural signal may be an important marker for the valuation of prosocial outcomes (see also Morelli *et al.,* 2018). Finally, we observed several separable activation patterns in social brain regions. Whereas TPJ was more strongly activated when gains deviated from the equity norm in favor of charity, insula and dACC were more strongly activated when gains deviated from the equity norm in favor of self. Together, the current results help to unravel individual sensitivities towards gaining for self and others.

## Supplementary Material

scan-19-102-File013
