# Supplementary material for: Neural reward related-reactions to monetar gains for self and charity are associated with donating behavior in adolescence
Source: Soc Cogn Affect Neurosci. 2020 Mar 12;15(2):151–63. doi: 10.1093/scan/nsaa027 (PMC7304510; doi:10.1093/scan/nsaa027)
Supplement: scan-19-102-File013 [file scan-19-102-file013.docx]

**Supplementary material A: List of available charities in fMRI task**

De Hartstichting

Artsen Zonder Grenzen

KWF Kankerbestrijding

CliniClowns

UNICEF

Dierenbescherming

Warchild

Greenpeace

WWF

**Supplementary file B: Activity in NAcc for each outcome condition**

**Figure B1.** Figure displays average parameter estimates of BOLD response of all modeled events.

**Supplementary File C: Timeseries of nucleus accumbens**


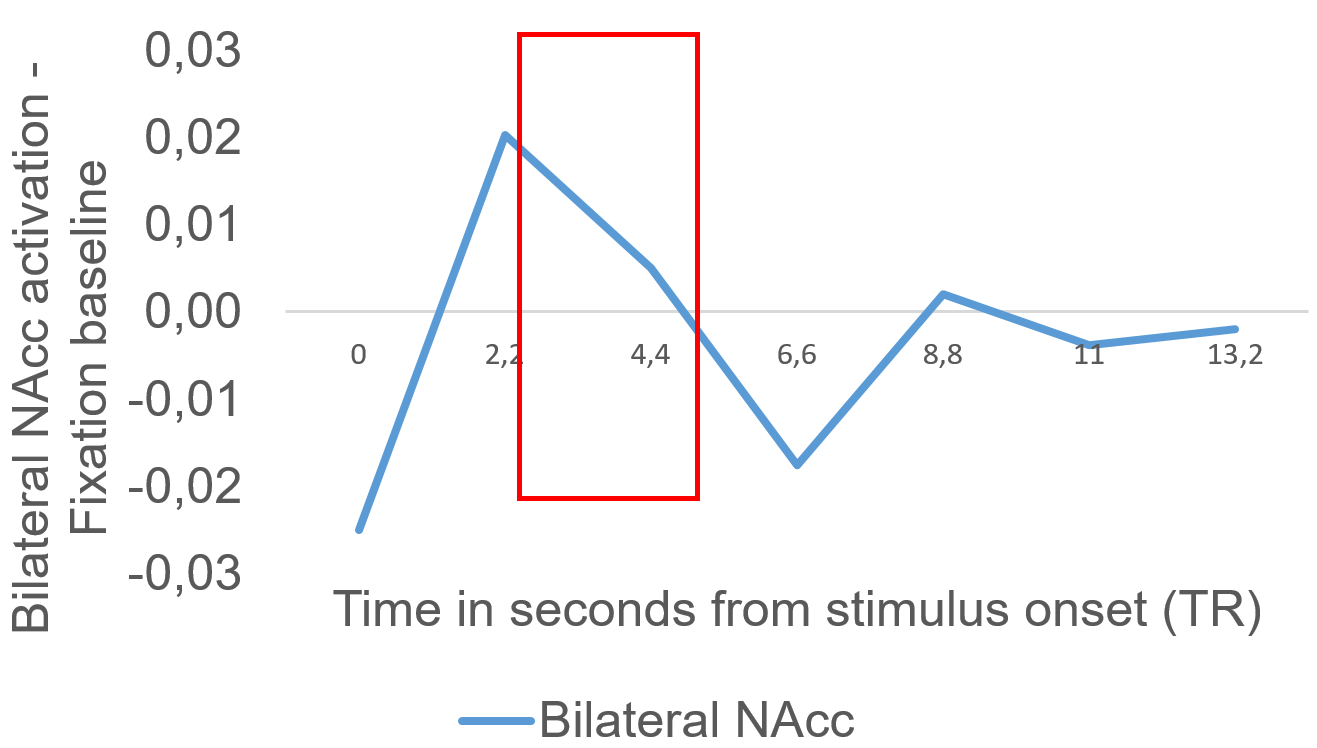


**Figures C1.** This figure shows the time series of activation in nucleus accumbens after stimulus onset. The window in red is the timeframe starting at the moment in time when the feedback is fully visible to the participant (2550 ms after stimulus onset; frame 7/14 of the opening animation), and ending at the offset of the feedback screen (5000 ms after stimulus onset).


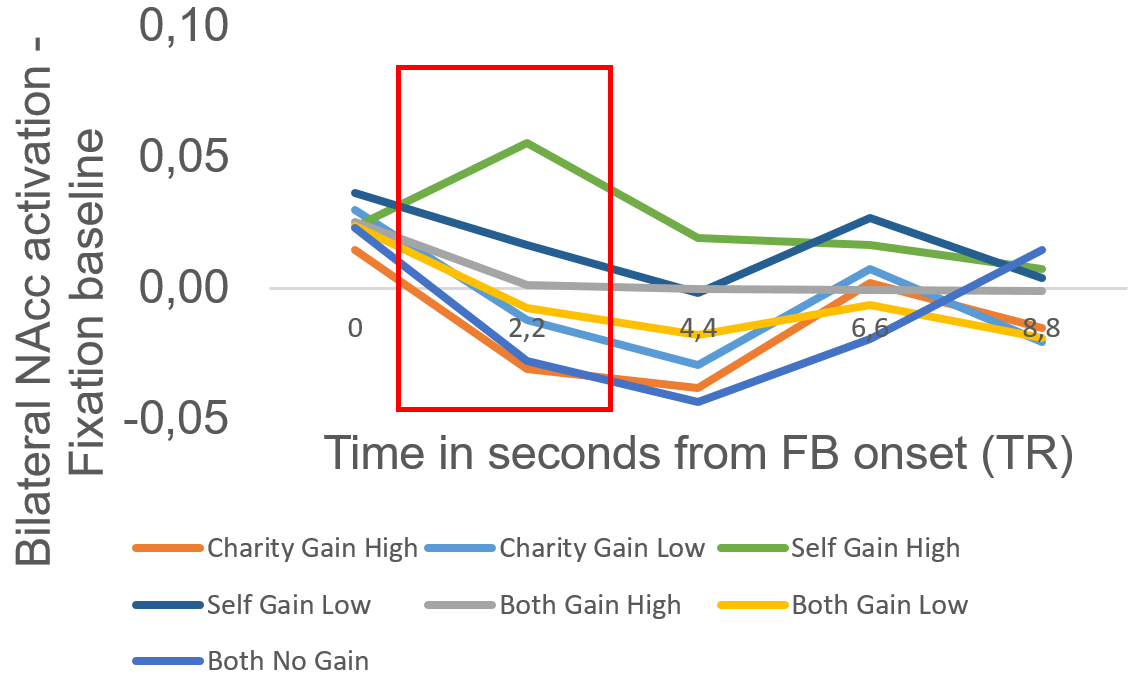


**Figures C2.** This figure shows the time series of activation in nucleus accumbens after feedback onset, shown separately for each of the seven conditions. The window in red is the timeframe starting at the moment in time when the feedback is fully visible to the participant (350 ms after FB onset), and ending at the offset of the feedback screen (3000 ms after FB onset), matching the window in red in Figure E1.

**Supplementary File D:** Scatterplots for self/charity enjoyment ratings and donation behavior

**
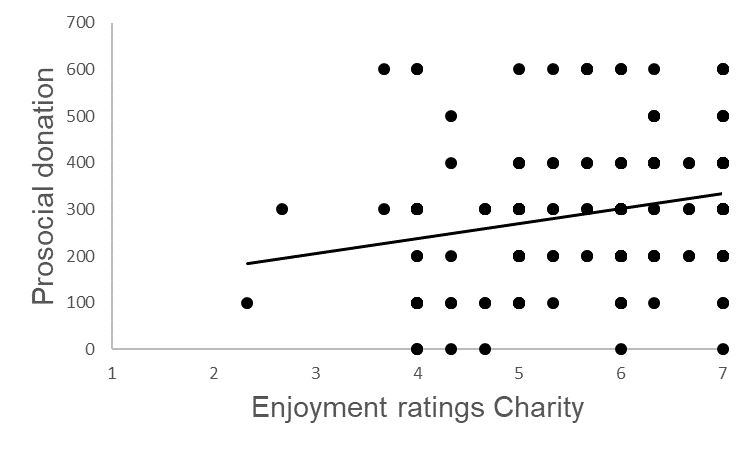
**

**Figure D1.** Visualization of correlation between enjoyment ratings for charity on the x-axis and donation behavior on the y-axis, r(160) = .234, *p < .01*

**
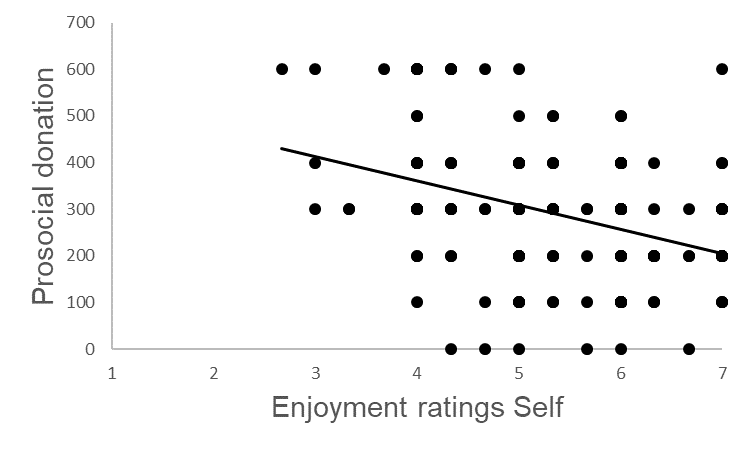
**

**Figure D2.** Visualization of correlation between enjoyment ratings for self on the x-axis and donation behavior on the y-axis. r(160) = .234, *p < .01*

**Supplementary material E:** Versions of respective tables 1 and 3 with parameter values.

Table E1 (table 1 in MS). *Correlations between self-reported empathic concern (IRI-EC), perspective taking (IRI-PT), enjoyment of self-gains and charity-gains, importance of charity, knowledge of charity, and donation behavior in the donating task. Degrees of freedom = 160. Significance values are displayed for each correlation. Significant p-values (p < .05) are flagged with an asterisk.*

|  |  |  | Perspective taking | Self- gains | Charity- gains | Importance | Knowledge | Donation  behavior | Age |
| --- | --- | --- | --- | --- | --- | --- | --- | --- | --- |
|  | Empathic Concern | Pearson Correlation | .307 | -.026 | .030 | .264 | .108 | .118 | -.103 |
|  |  | Significance | <.001* | .740 | .710 | .001* | .172 | .138 | .195 |
|  | Perspective taking | Pearson Correlation |  | -.082 | -.041 | -.097 | -.026 | .061 | .306 |
|  |  | Significance |  | .302 | .605 | .224 | .741 | .447 | <.001* |
|  | Self-gains | Pearson Correlation |  |  | .430 | .046 | .026 | -.364 | -.049 |
|  |  | Significance |  |  | <.001* | .564 | .746 | <.001* | .538 |
|  | Charity-gains | Pearson Correlation |  |  |  | .271 | .198 | .234 | -.102 |
|  |  | Significance |  |  |  | .001* | .012* | .003* | .199 |
|  | Importance | Pearson Correlation |  |  |  |  | .350 | .191 | -.290 |
|  |  | Significance |  |  |  |  | <.001* | .016* | <.001* |
|  | Knowledge | Pearson Correlation |  |  |  |  |  | .066 | -.084 |
|  |  | Significance |  |  |  |  |  | .408 | .291 |
|  | Donation Behavior | Pearson Correlation |  |  |  |  |  |  | .129 |
|  |  | Significance |  |  |  |  |  |  | .105 |

Table E2 (table 3 in MS). *Correlations between donation behavior and activation in Self Gain - Both No Gain, Both Gain - Both No Gain, and Charity Gain - Both No Gain. Degrees of freedom = 160. Significance values are displayed for each correlation. Significant p-values are flagged with an asterisk.*

|  |  |  | NAcc Self Gain | NAcc Both Gain | NAcc Charity Gain |
| --- | --- | --- | --- | --- | --- |
|  | Donation Behavior | Pearson Correlation | -.175 | -.068 | .102 |
|  |  | Significance | .027* | .393 | .197 |
|  | NAcc Self Gain | Pearson Correlation |  | .625 | .537 |
|  |  | Significance |  | <.001* | <.001* |
|  | NAcc Both Gain | Pearson Correlation |  |  | .615 |
|  |  | Significance |  |  | <.001* |

**Supplementary file F: ROI analyses, bilateral insula, right TPJ, and dACC.**

As exploratory analyses, we examined correlations between age, donation behavior and neural activity in the ROIs of the dACC, bilateral insula, and TPJ. We averaged across magnitudes as there was no main effect of magnitude nor an interaction with magnitude in our whole brain analyses.

**F1. Left Insula**

As can be seen in Table F1, there were negative relations between age and activity in the left insula in the Both Gain > Both No Gain. That is, older participants showed less activity in the left insula when gaining for both self and charity. No relation between neural activity and age was observed for Charity Gain > Both No Gain or Self Gain > Both No Gain. There was no significant relation to donation behavior.

Table F1. *Correlations between age, donation behavior, and activation in Self Gain - Both No Gain, Both Gain - Both No Gain, and Charity Gain - Both No Gain in Left Insula. Significant correlations are flagged at p < .05 with ‘*’ and at p* *< .001 with ‘**’.*

|  |  | Donation Behavior | L Insula Self Gain | L Insula Both Gain | L Insula Charity Gain |
| --- | --- | --- | --- | --- | --- |
|  | Age | .129 | -.102 | -.271** | -.136 |
|  | Donation Behavior |  | -.026 | -.068 | -.010 |
|  | L Insula Self Gain |  |  | .613** | .644** |
|  | L Insula Both Gain |  |  |  | .661** |

**F2. Right Insula**

As can be seen in Table F2, there were negative relations between age and activity in the right insula in the Both Gain > Both No Gain and Self Gain > Both No Gain. That is, older participants showed less activity in the right insula when gaining for both self and charity, and when gaining for self only. No relation between neural activity and age was observed for Charity Gain > Both No Gain.

Table F2. *Correlations between age, donation behavior, and activation in Self Gain - Both No Gain, Both Gain - Both No Gain, and Charity Gain - Both No Gain in Right Insula. Significant correlations are flagged at p < .05 with ‘*’ and at p* *< .001 with ‘**’.*

|  |  | Donation Behavior | R Insula Self Gain | R Insula Both Gain | R Insula Charity Gain |
| --- | --- | --- | --- | --- | --- |
|  | Age | .129 | -.167* | -.216** | -.140 |
|  | Donation Behavior |  | .015 | -.011 | .065 |
|  | R Insula Self Gain |  |  | .594** | .610** |
|  | R Insula Both Gain |  |  |  | .615** |

**F3. Right temporo parietal junction (R TPJ)**

Finally, as can be seen in Table F3, there were no relations between age and activity in the right TPJ.

Table F3. *Correlations between age, donation behavior, and activation in Self Gain - Both No Gain, Both Gain - Both No Gain, and Charity Gain - Both No Gain in Right TPJ. Significant correlations are flagged at p < .05 with ‘*’ and at p* *< .001 with ‘**’.*

|  |  | Donation Behavior |  | R TPJ Self Gain | R TPJ Both Gain | R TPJ Charity Gain |
| --- | --- | --- | --- | --- | --- | --- |
|  | Age | .129 |  | .016 | -.049 | .056 |
|  | Donation Behavior |  |  | .011 | -.039 | -.060 |
|  | R TPJ Self Gain |  |  |  | .607** | .657** |
|  | R TPJ Both Gain |  |  |  |  | .730** |

**F4. Dorsal anterior cingulate cortex (dACC)**

As can be seen in Table F4, there were no significant relations between age and activity in the dACC.

Table F4. *Correlations between age, donation behavior, and activation in Self Gain - Both No Gain, Both Gain - Both No Gain, and Charity Gain - Both No Gain in dACC. Significant correlations are flagged at p < .05 with ‘*’ and at p* *< .001 with ‘**’.*

|  |  | Donation Behavior | dACC Self Gain | dACC Both Gain | dACC Charity Gain |
| --- | --- | --- | --- | --- | --- |
|  | Age | .129 | -.013 | -.127 | -.070 |
|  | Donation Behavior |  | -.055 | .020 | -.026 |
|  | dACC Self Gain |  |  | .593** | .601** |
|  | dACC Both Gain |  |  |  | .663** |

**Supplementary File G: Exploratory age analyses for NAcc**

As can be seen in both Table G1 and Figure G2 (A & B), there were negative relations between age and activity in the Self Gain > Both No Gain, and Both Gain > Both No Gain. That is, older participants showed less activity in the ventral striatum when gaining for self or when gaining for both self and charity. No relation between neural activity and age was observed for Charity Gain > Both No Gain.

Table G1.

*Correlations between age, and activation in NAcc in the Self Gain - Both No Gain, Both Gain - Both No Gain, and Charity Gain - Both No Gain contrasts. Significant correlations are flagged at p < .05 with ‘*’ and at p* *< .001 with ‘**’.*

|  |  | NAcc Self Gain | NAcc Both Gain | NAcc Charity Gain |
| --- | --- | --- | --- | --- |
|  | Age | -.177* | -.161* | -.044 |
|  | NAcc Self Gain |  | .625** | .537** |
|  | NAcc Both Gain |  |  | .615** |

B

A

**Figure G2.** Relations are displayed between activation in Self Gain – Both No Gain and age (A), Both Gain – Both No Gain and age (B).
